# Effects of a Digital Health Intervention for Adults With Type 2 Diabetes Mellitus on Health Care Resource Use and Health Care Charges in the United States: Retrospective Cohort Study

**DOI:** 10.2196/67320

**Published:** 2025-11-17

**Authors:** Laura Wilson, Daniel Malone, Praveen Potukuchi, Edward Jonathan Han-Burgess, Keni C S Lee, Alison Edwards, Xinyan Yu, Nita Thingalaya, Felix Lee, Diana Brixner

**Affiliations:** 1 Sanofi Morristown, NJ United States; 2 Department of Pharmacotherapy College of Pharmacy University of Utah Salt Lake City, UT United States; 3 Sanofi Cambridge, MA United States; 4 Sanofi Reading United Kingdom; 5 Symphony Health ICON plc Blue Bell, PA United States

**Keywords:** digital health, type 2 diabetes mellitus, self-management, glycemic control, diabetic comprehensive care, health economic analysis, health care resource use, health care charges, economic burden

## Abstract

**Background:**

Type 2 diabetes mellitus (T2DM) is a chronic disease that requires management of blood glucose. According to previous studies, the Dario Digital Diabetes Solution (DDS) is a nonprescription digital health intervention with a smartphone app that has been shown to improve blood glucose control in adults with T2DM.

**Objective:**

This study aims to investigate the effects of DDS on health care resource use (HCRU) rates, charges, and estimated costs for adults with T2DM.

**Methods:**

In this retrospective cohort study, patient-level claims data of adults with T2DM were obtained from the Symphony Health Integrated Dataverse, a database containing both inpatient and outpatient claims, including diagnoses and procedures. Using exact and propensity score matching, DDS users and nonusers were matched in a 1:3 ratio. For the primary outcome measure (all-cause HCRU rates, defined as inpatient hospitalization and emergency room visits) and secondary outcome measures (all-cause outpatient visit rates, all-cause HCRU charges, and diabetes mellitus–related HCRU rates and charges), baseline, follow-up, and changes in values were summarized using descriptive statistics, and a multivariable generalized linear model or a 2-part model (including a generalized linear model) was applied. Additional exploratory outcome measures were analyzed. In a sensitivity analysis, a cost-to-charge ratio was calculated and applied to medical claims to estimate medical costs.

**Results:**

Following matching, cohorts consisted of 2445 DDS users and 7334 nonusers with similar demographic and baseline characteristics. The all-cause HCRU event rate was 9.3% lower in DDS users compared with nonusers at the 12-month follow-up from the index date. The mean number of events was estimated to be significantly lower in DDS users (0.48 per patient per year [PPPY]; 95% CI 0.44-0.52) than nonusers (0.52 PPPY; 95% CI 0.50-0.55), resulting in an incidence rate ratio of 0.91 (*P*=.04). Inpatient hospitalization was 23.5% lower in the DDS user cohort compared with the nonuser cohort, with emergency room visit and outpatient visit rates being similar across both cohorts. DDS users were numerically less likely to incur all-cause HCRU charges than nonusers (odds ratio 0.91, 95% CI 0.82-1.01; *P*=.07). All-cause HCRU charges were 26% lower for DDS users than for nonusers (US $ 12,552 PPPY savings; *P<*.001). When applying the cost-charge-ratio to the charges, the total estimated cost saving for DDS users was US $5077, of which US $4513 PPPY was attributed to all-cause HCRU and US $564 to all-cause office visits.

**Conclusions:**

In this retrospective cohort study of adults in the United States with T2DM, DDS users were found to have lower all-cause HCRU rates than nonusers, driven by significantly lower inpatient hospitalization rates (*P*<.001). All-cause HCRU charges and estimated costs were shown to be lower for DDS users compared with nonusers.

## Introduction

### Background

Diabetes mellitus (DM) is a group of metabolic diseases characterized by insulin insufficiency and insulin resistance, often resulting in hyperglycemia [1]. In the United States, the estimated prevalence of diagnosed DM in 2017 was 28.7 million people [2]. Approximately 90% to 95% of people in the United States who have DM have type 2 DM (T2DM), which often develops in people aged >45 years but is increasingly being diagnosed in younger people [3]. The estimated direct medical costs associated with DM were US $307 billion in 2022, an increase from US $237 billion in 2017 [4,5]; 31% of those costs were attributed to hospital inpatient care and 11% to office visits. Medical complications are key drivers of the direct medical costs of treating patients with T2DM [5].

The long-term effects of DM include damage to blood vessels, which can result in macrovascular (eg, myocardial infarction, stroke, and cerebrovascular disease) and microvascular complications (eg, retinopathy, nephropathy, and neuropathy) [6]. DM is a chronic condition with no cure, but through careful management, diabetic complications can be delayed, managed, or prevented [7-9]. Poor glycemic control, a measure of DM management, is associated with an increased incidence of complications and reduced quality of life [8]. In the United States, approximately 50% of the people with T2DM achieve glycemic control targets—a proportion that has declined over the past decade despite the availability of new therapeutic options [10]. Among people with T2DM, therapeutic inertia is common even after patients have initiated basal insulin [11,12]. An increased focus on disease management can have positive impacts on clinical outcomes. Self-management education and structured self-monitoring and intervention by nonphysician providers have been shown to improve glycated hemoglobin (HbA_1c_) levels [13-15]. These improvements in HbA_1c_ have been shown to translate into reductions in health care costs. For patients with T2DM, a 1% reduction in HbA_1c_ has been associated with a 13% reduction in DM-related total health care costs [16].

There is growing evidence that digital interventions can play a role in addressing the challenges associated with reducing the clinical and economic burden associated with T2DM. Digital technologies that allow for personalized intervention have been developed to improve DM care management [17], offering people with DM new opportunities to better manage their disease and improve their health-related quality of life [18,19]. A qualitative study by Turnbull et al [19] exploring the attitudes of patients with T2DM toward digital technologies highlighted the benefits patients felt, including better understanding and control of their condition, empowerment in interactions with health care professionals, and avoiding stigma associated with T2DM.

### Objective

Dario Digital Diabetes Solution (DDS) is a nonprescription digital health intervention with a smartphone app that combines remote self-monitoring of blood glucose, disease education, and data visualization to facilitate behavior change. The use of DDS has been associated with improvements in blood glucose control in adults with T2DM [20]. Better management of T2DM, through the use of a digital intervention, has the potential to decrease health care resource use (HCRU), resulting in savings for health care systems. The overall objective of this study was to investigate the effects of DDS on HCRU, charges, and estimated costs for adults with T2DM.

## Methods

### Data and Study Cohorts

This US retrospective cohort study included patients with T2DM who first registered for DDS between January 1, 2017, and April 30, 2021, or patients with T2DM with a medical encounter first recorded within the same period in the nonuser cohort. The study also included a 12-month follow-up period for all patients. The perspectives adopted are primarily those of health care payers, with the goal of providing evidence on the impact of DDS use versus nonuse on health care delivery and costs.

This study used real-world data relating to patient health status and the delivery of health care. The data source contained data from DDS users that were deidentified and tokenized with SynomaID, a unique patient identifier, linked with the Symphony Health Integrated Dataverse (IDV). The IDV database contains approximately 286 million individuals, 10,000 tracked health plans, and >14 years of historical data, which covers approximately 92% of prescriptions dispensed in the United States and its territories and about 60% of US medical claims. The IDV contains both inpatient and outpatient claims, including diagnoses and procedures. Medical claims included in the IDV provide the total billed amounts (charges) and not the paid amounts. Paid pharmacy claims were obtained directly from pharmacy data and included payer and consumer cost-sharing amounts. Demographic data, including age, sex, and geographic region as well as payer type, facility type, and health care provider specialty, are included in the IDV.

This study compared patients with T2DM who were DDS users with nonusers. For both cohorts, adults (aged ≥18 y) with T2DM were identified from medical claims using *International Classification of Diseases, Ninth Revision* or *International Classification of Diseases, 10th Revision* codes. Additional inclusion criteria were receiving 1 or more antidiabetic medications within 365 days before the index date, access to care for 365 days before index date and 365 days after index date (confirmed by evidence of 1 or more medical or hospital and 1 pharmacy claim within both periods, in lieu of continuous enrollment information), and 1 or more inpatient or 2 or more outpatient visits more than 30 days apart during the baseline period. Exclusion criteria for both the DDS user and nonuser cohorts were individuals aged <18 years, use of continuous glucose monitoring during the study period, diagnosis of type 1 DM or gestational diabetes, and hospital discharge without physician consent. In addition, DDS users had to have an initial DDS registration between January 1, 2017, and April 30, 2021.

The index date for DDS users was the date of first DDS registration within the study period. For DDS nonusers, a simple random sampling technique was used to assign each DDS nonuser to a quarter of the study period in which the DDS nonuser had 1 or more medical claims. The index date for the nonuser was then defined as the first date of any medical encounter in the assigned quarter. For both cohorts, the baseline period was defined as 365 days before the index date, and the follow-up period was 365 days after the index date, to allow the impact of the DDS to be measured over the first year after registration.

To address potential bias in the study sample, analysis was conducted on matched cohorts selected using exact and propensity score matching methodologies with sequential matching at a ratio of 1:3 using SAS Viya (version 4; SAS Institute). The initial cohort of DDS nonusers was refined to a subset of patients that matched 1 or more DDS users based on all exact-matching criteria: the quarter of the study period in which the index date falls, sex, payer type, and baseline antidiabetic medication (oral antidiabetic drug only; insulin only; and any other, including combinations). The DDS nonusers were also matched through a propensity score matching process without replacement to achieve a ratio of 1 DDS user to 3 nonusers. In addition to the exact score matching criteria, independent variables included in the propensity score model were age, race or ethnicity, region, Quan-Charlson Comorbidity Index score, other comorbidities that are risk factors for diabetes (hypertension, hyperlipidemia, anemia, and depression), comedications that may impact blood glucose levels (iron, antihypertensives, dyslipidemia medication, antidepressants and anxiolytics, steroids, and proton pump inhibitors), and baseline HCRU. A greedy nearest neighbor matching algorithm without replacement matched users with nonusers based on the logit of propensity score using a caliper equal to 0.2 of the SD of the logit of the propensity score. After matching, the balance of covariates between matched groups was assessed by calculating the standardized mean differences, which were less than 0.1. Matching was performed in a similar manner to that described in a study (Kerr et al, unpublished data, September 2025).

### Outcome Measures

All analyses were conducted under the intention-to-treat assumption, as no data were available to indicate disenrollment from the DDS. After access to care criteria were applied, missing values for the variables derived from claims (eg, diagnoses, prescriptions, use, and cost) were considered 0 and nonmissing. Access to care criteria ensured that any lack of information was not due to a lack of data, and where there were any missing claims data, it was due to the service not occurring. Missing data were quantified in terms of the number of unique patients with missing data, and values were not imputed. For all outcome measures, charges and costs were adjusted to 2022 US dollars based on the consumer price index (Multimedia Appendix 1) [21].

For the primary outcome measure, a composite end point of all-cause HCRU event rates included inpatient hospitalizations and emergency room (ER) visits [22]. Baseline, follow-up, and change in all-cause HCRU rates were summarized using descriptive statistics as well as per person per year (PPPY) measure. A subgroup analysis was performed based on the use of blood glucose test strips.

Secondary outcome measures were all-cause HCRU outpatient visit rates, all-cause HCRU charges, and DM-related HCRU rates and charges. All-cause HCRU outpatient visit rates included physician office visits and other outpatient visits, the latter being medical claims occurring in the outpatient setting, according to the place of service code (hospital outpatient, other facility, or location) that do not indicate a medical clinic or physician’s office. All-cause HCRU charges are a composite measure of the charges associated with specific points of service (ie, inpatient hospital stay and ER visit). DM-related HCRU rates and charges included hospitalization, ER visits, physician office visits, and other outpatient visits where the primary diagnosis was recorded as T2DM.

Exploratory outcome measures included medical or pharmacy claims, pharmacy costs, readmission rates (all cause, 30 day, and DM related), and length of stay among patients with 1 or more all-cause inpatient events as well as comparing all-cause HCRU rates and charges by DDS user engagement activity frequency. DDS user engagement activity was measured in active days (ie, the number of days when a user performed any 1 of the 10 engagement activities during the follow-up period). Engagement activities included food logging (eg, carbohydrate counting and meal photos), inputting insulin dose, interacting with a coach, measuring blood glucose, measuring blood pressure, measuring weight, recording physical activity, sharing logbook, tagging (timing of blood glucose measurement and meal type), and reading an educational article. Unless otherwise stated, the assessments were made from baseline through 12 months of follow-up after the index date.

IDV medical claims do not include the paid amount; therefore, submitted charges were used to compare the economic impact of DDS. Remittance data can provide information on physician reimbursement by the plan and patient out-of-pocket costs. In this study, the remittance data were linked with IDV medical claims data to find or estimate the cost. Not all IDV medical claims can be matched to remittance data. Therefore, in a sensitivity analysis, the cost-to-charge ratio (CCR) was calculated from remittance data and applied to the IDV medical claims that lacked data on actual amounts paid to estimate costs associated with the treatment of DDS users and nonusers and any potential differences. This methodology was applied to medical claims only. Details of the calculation, verification, and application of the CCR are outlined in the Multimedia Appendix 2.

### Statistical Analyses

Analyses were calculated using Oracle SQL Developer (version 18.4.0.376; Oracle Corporation) and WPS Workbench (version 4.0.0.0.6435; Altair Engineering). All demographic and baseline clinical characteristics and HCRU rates and charges were described separately for matched DDS users and nonusers using means, SDs, medians, IQRs, and minimum and maximum ranges for continuous variables and counts with percentages for categorical or binary variables. Balance between the 2 cohorts was calculated as standardized mean differences, before and after matching (Multimedia Appendix 3).

To analyze the primary outcome measure (all-cause HCRU rates), a multivariable generalized linear model (GLM) with a negative binomial distribution was used to analyze the data. Incidence rate ratio (IRR) was calculated, and statistical significance was determined using Wald chi-square statistics.

Secondary outcome measures (all-cause office visits and other outpatient visits, DM-related hospitalization and ER visits, and office visits and other outpatient visits) were analyzed in a similar manner to the primary objective with a GLM and negative binomial distribution. The GLM specifications for each secondary outcome measure were determined based on descriptive statistics. In the analysis of the HCRU charges (all cause and DM related), a 2-part model was used, with a logistic regression model followed by GLM with a gamma distribution. In the 2-part model, the likelihood (odds ratio; OR) of DDS users vs nonusers incurring charges was determined first. Second, total charges PPPY in patients who had incurred charges were calculated.

For the exploratory outcome measures, total all-cause medical and pharmacy claim counts and all-cause pharmacy costs were described using descriptive statistics, reported as PPPY, as well as univariable and multivariable GLMs. Readmission analyses used a 2-part GLM to test the association between DDS and readmissions, where the first part of the model was a logistic regression model with a binary indicator of all-cause hospitalization versus no hospitalization, and the second part was readmission. When calculating length of stay, total all-cause inpatient days were included, and comparisons between the cohorts were made using a 2-sample, 2-tailed *t* test.

### Ethical Considerations

By definition this study did not involve direct interaction with human participants or the collection of identifiable personal data, and no compensation to any human participants was associated with this study. The analysis was a secondary use of deidentified data and conducted retrospectively using fully anonymized data. As such, no ethical approval was required or sought, in accordance with institutional and international guidelines on research involving human subjects.

## Results

### Cohort Demographics

**Figure 1:**
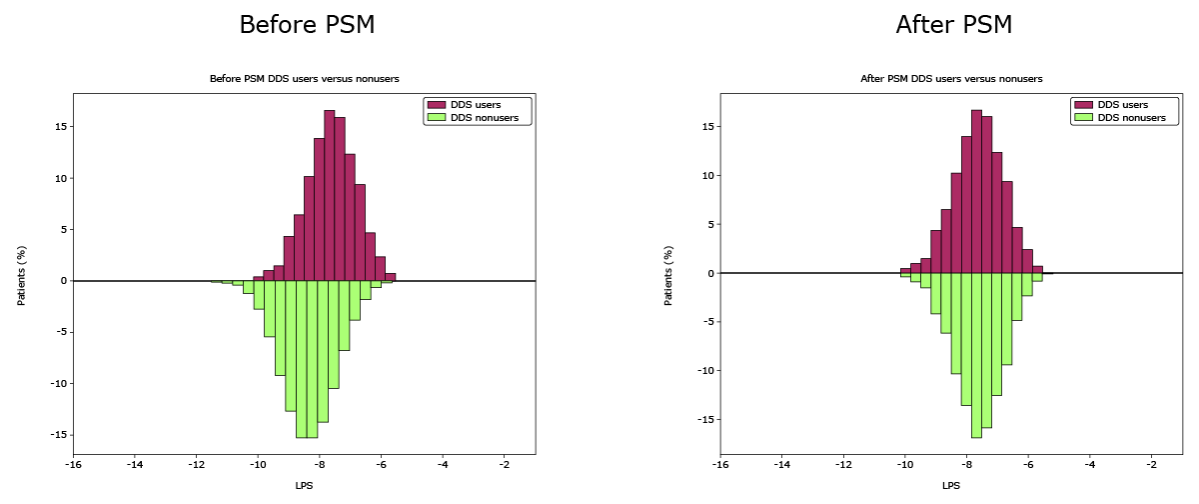
Logit propensity score (LPS) of Dario Digital Diabetes Solution (DDS) user and nonuser cohorts before and after propensity score matching (PSM).

**Figure 2:**
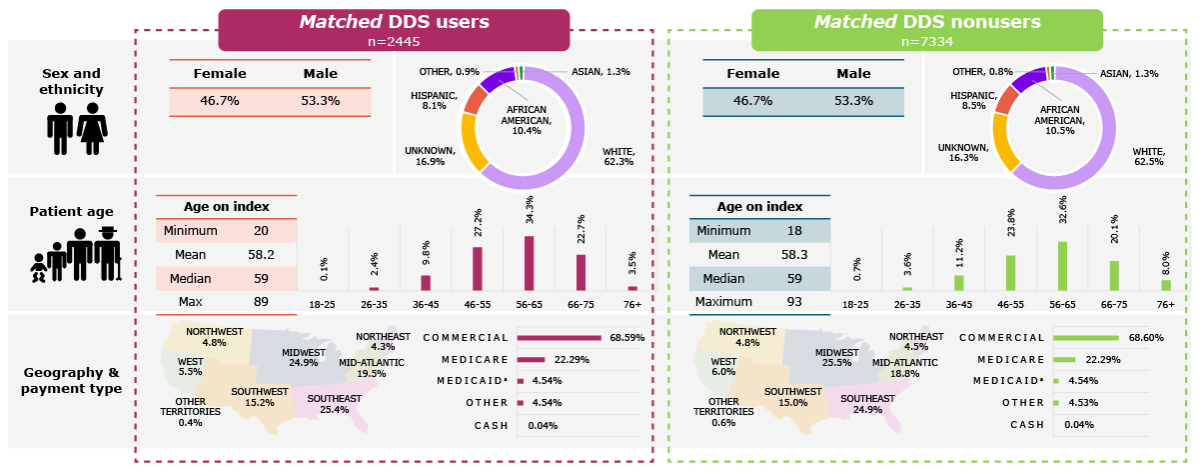
Demographics and baseline characteristics of the matched Dario Digital Diabetes Solution (DDS) user and nonuser cohorts. Description of race has been updated from Caucasian to White to more accurately reflect the population. *Medicaid or managed Medicaid.

Following exact and propensity score matching, the DDS user and nonuser cohorts contained 2445 and 7334 patients, respectively. The matched DDS user and nonuser cohorts had similar demographic and baseline characteristics (Figures 1 and 2; Table 1). In the matched cohorts, mean age was 58.2 (SD 10.6) years in the DDS user cohort and 58.3 (SD 12.5) years in the nonuser cohort. Most people in the matched DDS user and nonuser cohorts were White (1524/2445, 62.33% and 4584/7334, 62.50%, respectively), and most were covered by commercial payers (1677/2445, 68.59% and 5031/7334, 68.60%, respectively).

**Table 1:** Table1. Demographic and baseline characteristics of the matched Dario Digital Diabetes Solution (DDS) user and nonuser cohorts.

Demographic and baseline characteristics			DDS users (n=2445)		DDS nonusers (n=7334)		Standardized mean difference		
							Before matching		After matching
**Index year, n (%)**									
	2017	577 (23.6)		1730 (23.59)		—^a^		—	
	2018	604 (24.70)		1812 (24.71)		—		—	
	2019	491 (20.08)		1473 (20.08)		—		—	
	2020	569 (23.27)		1707 (23.28)		—		—	
	2021^b^	204 (8.34)		612 (8.34)		—		—	
**HbA_1c_^c^**									
	HbA_1c_ (%)	629 (25.73)		1830 (24.95)		—		—	
	Mean (SD)	8.20 (1.95)		7.49 (1.78)		—		—	
**Antidiabetic medication, n (%)**									
	Insulin only	175 (7.16)		524 (7.14)		—		—	
	OAD^d^ only	1297 (53.05)		3891 (53.05)		—		—	
	Any combination or other injectable	973 (39.8)		2919 (39.80)		—		—	
Quan-Charlson comorbidity score, mean (SD)			1.50 (1.50)		1.55 (1.59)		—		—
**Comorbidities, n (%)**									
	Blood anemia	110 (4.5)		309 (4.21)		0.128		–0.012	
	Hypertension	1466 (59.96)		4451 (60.69)		0.104		0.015	
	Hyperlipidemia	1278 (52.27)		3908 (53.29)		0.042		0.020	
	Depression	304 (12.43)		889 (12.12)		–0.069		–0.010	
**Concomitant medication, n (%)**									
	Antidepressant or anxiolytic medications	1007 (41.19)		2986 (40.71)		–0.207		–0.010	
	Anemia medications	35 (1.43)		114 (1.55)		0.064		0.009	
	Antihypertensive medications	1924 (78.69)		5846 (79.71)		0.102		0.026	
	Steroid medications	728 (29.78)		2157 (29.41)		–0.033		–0.008	
	Dyslipidemia medications	1639 (67.03)		4962 (67.66)		0.079		0.013	
	PPI^e^ medications	682 (27.89)		2031 (27.69)		0.021		–0.004	
**Events, mean (SD)**									
	Inpatient	0.22 (0.78)		0.19 (0.55)		–0.111		0.030	
	ER^f^	0.37 (1.17)		0.35 (0.88)		–0.092		0.016	
	Clinic or physician’s office	6.31 (14.24)		6.17 (14.91)		0.017		0.010	
	Other outpatient	3.57 (6.27)		3.47 (7.98)		–0.123		0.008	

^a^Not applicable.

^b^2021 is a partial year.

^c^HbA_1c_: glycated hemoglobin.

^d^OAD: oral antidiabetic drug.

^e^PPI: proton pump inhibitor.

^f^ER: emergency room.

### Primary Outcome Measure

**Figure 3:**
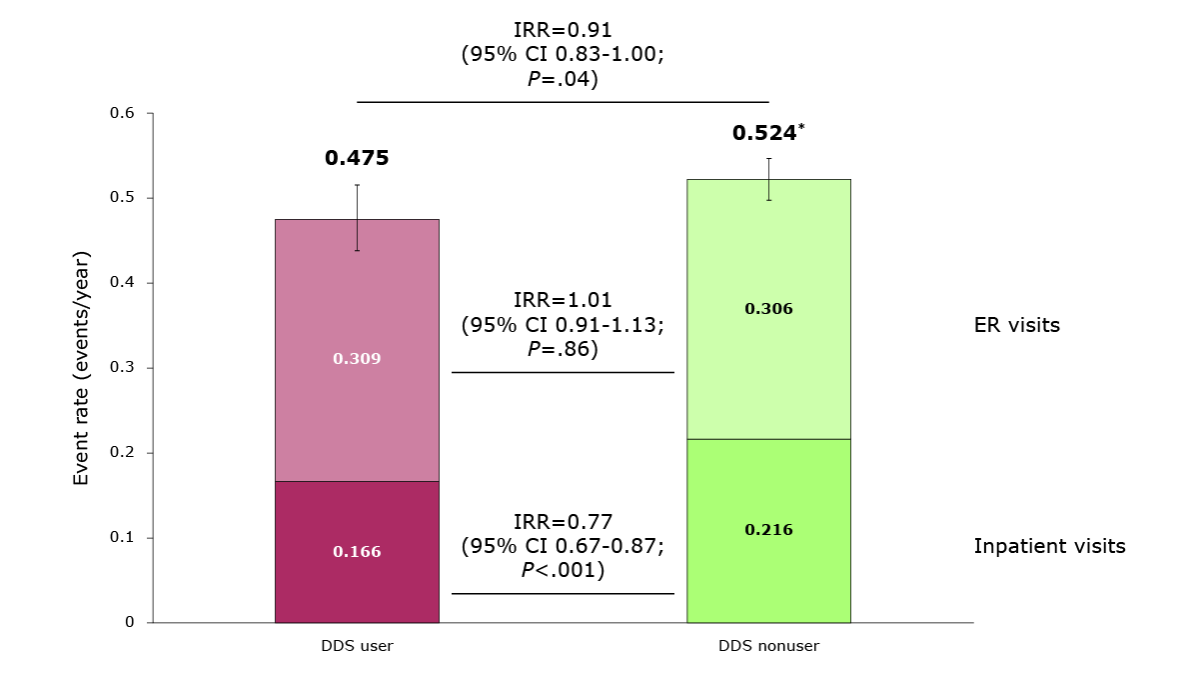
All-cause health care resource use (HCRU) rate (inpatient hospitalizations and emergency room [ER] visits) at 365 days. *Values do not sum due to rounding. DDS: Dario Digital Diabetes Solution; IRR: incidence rate ratio.

The all-cause HCRU event rate was 9.3% lower for DDS users than nonusers in the 12-month follow-up from the index date (Figure 3). The GLM estimated that the mean number of events PPPY for DDS users was 0.48 (95% CI 0.44-0.52), and for nonusers it was 0.52 (95% CI 0.50-0.55), with an IRR of 0.91 (*P*=.04). This difference was driven by a 23.5% lower inpatient hospitalization rate in DDS users than in nonusers, with ER visit rates being similar across both cohorts. Mean all-cause hospitalization rates were 0.17 (95% CI 0.15-0.19) and 0.22 (95% CI 0.20-0.23) events PPPY for DDS users and nonusers, respectively, with an IRR of 0.77 (*P*<.001). Mean all-cause ER visit rates were 0.31 (95% CI 0.28-0.34) and 0.31 (95% CI 0.29-0.32) events PPPY for DDS users and nonusers, respectively, with an IRR of 1.01 (*P*=.86).

A smaller proportion of DDS users used blood glucose test strips at baseline compared with the nonuser cohort (806/2445, 32.97% vs 2901/7334, 39.56%; *P*<.001). In these subgroups, the mean events PPPY in the follow-up period were nominally lower in the DDS user cohort than in the nonuser cohort, but the difference was not statistically significant (IRR=0.87, 95% CI 0.74-1.02; *P*=.08).

### Secondary Outcome Measures

All-cause outpatient rates were similar between the 2 cohorts at follow-up, with an estimated rate of 8.97 PPPY for DDS users compared with 8.83 PPPY for nonusers, which translates to an IRR of 1.02 (95% CI 0.97-1.06; *P*=.47). There was a statistically significant increase in all-cause office visits at 12 months after index visit in the DDS user cohort compared with the nonuser cohort (*P*=.002). The estimated mean numbers of office visits PPPY for the DDS user and nonuser cohorts were 5.21 (95% CI 4.97-5.46) and 4.78 (95% CI 4.65-4.91), respectively (IRR=1.09; *P*=.002). Conversely, the rate of other outpatient visits PPPY at follow-up was significantly lower in the DDS user cohort compared with the nonuser cohort. The PPPY rate of other outpatient visits was 3.17 and 3.41 among the DDS user and nonuser cohorts, respectively, with an IRR of 0.93 (95% CI 0.87-1.00; *P*=.04)

The odds of a DDS user incurring all-cause HCRU charges were lower than nonusers, though not significantly (OR 0.91, 95% CI 0.82-1.01; *P*=.07). All-cause HCRU charges were 26% lower for DDS users than for nonusers (US $35,719 and US $48,272, respectively; US $12,552 PPPY savings; *P*<.001). DDS users were more likely to incur all-cause office visit charges than nonusers (*P*=.04); however, DDS users who incurred charges had 19% lower all-cause office visit charges than nonusers (US $7444 and US $9234, respectively; US $1790 PPPY savings; *P*<.001). In sensitivity analyses, when applying the aggregated CCR to the charges, the estimated costs in individuals who incurred more than US $0 all-cause HCRU charges were US $12,843 and US $17,356 for all-cause HCRU (inpatient and ER) for DDS users and nonusers, respectively. This resulted in an estimated cost saving of US $4513 PPPY. Similarly, in individuals who incurred more than US $0 all-cause HCRU charges, estimated costs for all-cause office visits were US $2345 for DDS users and US $2909 for nonusers, an estimated cost saving of US $564 PPPY. Total estimated cost savings of DDS users were US $5077 PPPY from medical claims.

Focusing on DM-related HCRU, it was found that the proportions of DDS users and nonusers who had DM-related events were low (3.1% and 3%, respectively). There were no significant differences in DM-related inpatient and ER inpatient only or ER-only visit rates between the DDS users and nonusers. The PPPY rate of inpatient and ER visits was 0.031 and 0.033 in the DDS user and nonuser cohorts, respectively, with an IRR of 0.94 (95% CI 0.70-1.26; *P*=.68). Separately, the rate of inpatient and ER visits was 0.019 for both cohorts (IRR=0.99, 95% CI 0.70-1.41; *P*=.96) and 0.012 and 0.014 for DDS users and nonusers, respectively (IRR=0.837, 95% CI 0.53-1.31; *P*=.44).

The number of DM-related outpatient visits was statistically significantly higher among DDS users compared with nonusers. The DDS user cohort had a mean outpatient visit rate of 1.45 PPPY, while the nonuser cohort was associated with a 1.17 PPPY rate, translating to an IRR of 1.24 (95% CI 1.16-1.32; *P*<.001). When considering visits to physicians specifically, the PPPY rate among DDS users was 0.87 compared with 0.69 for nonusers, with an IRR of 1.27 (95% CI 1.18-1.37; *P*<.001). Similarly, other outpatient visits had a reported rate of 0.47 and 0.37 PPPY for DDS users and nonusers, respectively, and an IRR of 1.27 (95% CI 1.12-1.45; *P*<.001).

The differences in the odds of incurring DM-related inpatient and ER visit charges (OR 0.98, 95% CI 0.75-1.29; *P*=.89), inpatient only charges (OR 1.01, 95% CI 0.79-1.54; *P*=.57), and ER only charges (OR 0.82, 95% CI 0.54-1.25; *P*=.35) were not significantly different between the cohorts. DDS users did have increased odds of incurring office visit costs (OR 1.27, 95% CI 1.14-1.41; *P*<.001), but when office visit charges were incurred, the costs were not significantly different between the 2 cohorts (DDS users: US $767, 95% CI US $724-US $813 vs DDS nonusers: US $764, 95% CI US $737-US $792; *P*=.91).

### Exploratory Outcome Measures

Analysis of the medical and pharmacy claim counts of DDS users and nonusers showed that there was no significant difference in all-cause medical claims or all-cause pharmacy claims at 12 months of follow-up. The mean number of medical claims per year was 12.2 for both DDS users and nonusers (IRR=1.00, 95% CI 0.96-1.05; *P*=.86), while the mean number of pharmacy claims was 42.5 in the DDS user cohort and 41.6 in the nonuser cohort (IRR=1.02, 95% CI 0.99-1.05; *P*=.15). After adjusting for baseline pharmacy costs, which were directly obtained from pharmacy claims data, there was a statistically significant increase in pharmacy costs paid by payers for DDS users compared with nonusers (DDS users: US $11,312, 95% CI US $10,786-US $11,837 vs DDS nonusers: US $10,005, 95% CI US $9702-US $10,309; *P*<.001). However, the difference in patient-paid pharmacy costs between the 2 cohorts was not statistically significant (DDS users: US $949, 95% CI US $815-US $1082 vs DDS nonusers: US $795, 95% CI US $718-US $872; *P*=.05).

At 12-month follow-up, 13.37% (327/2445) of the DDS users and 16.31% (1196/7334) of the nonusers had experienced 1 or more inpatient events. Readmission rates were also similar between the 2 cohorts. In the DDS user cohort 14.1% (46/327) of the patients who experienced an inpatient admission were readmitted compared with 18.56% (222/1196) of nonusers; this was not a statistically significant difference (*P*=.06). However, there was a statistically significant lower 30-day all-cause readmission rate for the DDS user cohort compared with nonusers (IRR=0.64, 95% CI 0.45-0.92; *P*=.01). When limited to DM-related admissions, the incidence rate of DM-related readmissions was also not significantly different, with 6% (3/50) of the DDS users experiencing readmission following DM-related admission compared with 4.5% (6/133) of the nonusers (*P*=.68). Mean inpatient length of stay was statistically significantly shorter for DDS users than nonusers (7.2 vs 8.8 days; *P*=.02).

Upon examining solely the DDS user cohort, the overall engagement (use of any 1 of the 10 DDS components) was associated with a 10.4% reduction in all-cause HCRU (inpatient and ER visit; for 100 days of engagement: IRR=0.90, 95% CI 0.83-0.97; *P*=.005). Being engaged with any of the 10 DDS components was associated with 14.8% decreased odds of incurring all-cause HCRU-related charges more than US $0 (inpatient and ER visit; for 100 days of engagement: OR 0.85, 95% CI 0.78-0.93; *P*<.001).

## Discussion

### Principal Findings

In this analysis of the DDS, the use of DDS was associated with lower all-cause HCRU rates (primary end point) compared with nonusers. One of the secondary end points, rates of all-cause outpatient visits, demonstrated similar rates between the 2 cohorts. All-cause HCRU charges (inpatient hospitalizations and ER visits) were incurred at a lower rate in the DDS user cohort compared with the nonuser cohort (although the difference was not significant). However, when charges were incurred, the value was significantly lower for the DDS user cohort versus the nonuser cohort. DM-related HCRU rates were similar between the cohorts, as were the DM-related HCRU charges.

Analysis of the all-cause office visits showed a significantly higher rate among DDS users compared with nonusers, which could be attributed to additional monitoring of patients. The increased monitoring associated with DDS could also trigger earlier health care professional intervention, as patients received more information about their health status. The relationship between DDS engagement and all-cause HCRU further supported this explanation, with greater DDS engagement being associated with a significant decrease in HCRU rates. In the context of fewer and briefer hospitalizations, more office visits can be seen as a positive outcome, especially if the charges associated with those visits are lower than for other similar patients.

Compared with DDS nonusers, DDS users had significantly shorter inpatient length of stay and significantly reduced 30-day readmission rates, both of which are indicative of improvements in quality of care. The importance of these improvements being associated with DDS use is reinforced by the emphasis placed on improvements in the effectiveness of care in the Healthcare Effectiveness Data and Information Set [23]. A reduction in readmissions and length of stay directly benefits all health care stakeholders; indirect benefits, such as reduced risk of hospital-acquired infections and their associated morbidity and costs, reduced time off work, and better productivity, may also be seen [24-27].

Although only charges were evaluated for HCRU, remittance data were available for medical claims, allowing for the calculation of a CCR, providing a payer perspective for these outputs. The estimated cost saving to payers associated with DDS use was US $5077 PPPY, which was calculated using the CCR based on medical claims data. Pharmacy costs, calculated directly from claims data, were US $1306 higher in DDS users compared with nonusers. In 2022, in the United States, patients with DM were estimated to incur average medical expenditures of approximately US $19,736 per year, approximately US $12,022 of which were attributed to DM, an increase in the costs estimated in 2017 of US $16,750 and US $9600, respectively [4,5]. Therefore, the estimated cost savings associated with DDS would likely contribute to meaningful reductions in the economic burden associated with T2DM. For comparison, a database analysis reported that a 1% reduction in HbA_1c_ was associated with US $429 and US $736 reductions in all-cause and DM-related total health care costs, respectively [16].

Most of the DM-related HCRU and charge measures did not show significant differences between the cohorts. The cause of this may be multifactorial. Overall, the rates of DM-related HCRU were low (DDS users: 3.1%; nonusers: 3%); therefore, detecting statistically significant differences was less likely. Although the DDS user and nonuser cohorts could be considered large, the study population was not powered for DM-related HCRU, which is a subset of all-cause HCRU and may not be able to detect small differences in DM-related HCRU. The protocol defined DM-related HCRU as any claim with a DM diagnosis code in the primary position only, which may have resulted in some DM-related events not being recognized due to the nature of the coding rank order. Focusing solely on DM-related HCRU may mean that the bigger picture of T2DM and its management is missed in terms of the whole-person effects and consequences of the disease. Moreover, the health system must pay the total cost of care for a person with T2DM and not just the costs associated with DM itself. There is also the possibility of differences in the types of medication provided to DDS users and nonusers based on the more frequent monitoring among DDS users.

The number of pharmacy claims did not differ significantly between DDS users and nonusers. DDS users had statistically significantly greater pharmacy costs paid by payers compared with nonusers; however, differences in patient-paid costs were not statistically significant. These findings are intriguing, and further research to determine the factors underlying the increase in costs without an increase in claim counts may be valuable. One potential explanation could be increased medication adherence among DDS users, leading to increased medication use and resulting in increased costs.

Observational studies of digital therapeutics are often conducted to inferior standards compared with premarket analysis, with deficiencies in demographic data reporting. These studies often exclude older adults and people not proficient in English, resulting in these studies not accurately reflecting the population [28]. In our analysis, comprehensive patient demographics of the matched cohorts were reported, and sourcing data from the IDV database reduced the bias toward younger adults and those proficient in English. Comparison of our analysis of DDS with a similar retrospective analysis of a remote digital diabetes management program in patients with DM contextualized the projected cost savings of this analysis. Whaley et al [29] estimated a cost saving of US $88 per patient per month (US $1056 PPPY), attributable to decreases in DM-related medical and office-based service spending. The study included only patients with commercial insurance, which skewed predominantly to those who were aged <65 years and therefore were not representative of the general US population. This is especially true given that 24.4% of people aged ≥65 years have been diagnosed with diabetes compared with only 3% of people aged between 18 and 44 years and 14.5% of the people aged between 45 and 64 years [30,31]. This analysis closely reflected the general US population, with more than 25% of the matched DDS user and nonuser cohorts being aged ≥65 years. In addition, patients in the analysis by Whaley et al [29] did not undergo a rigorous matching process, resulting in significant differences in age and Quan-Charlson Comorbidity Index between cohorts. Another study by Sweet et al [32] of a digital diabetes prevention program reported cost savings of US $1169, driven by fewer hospital admissions and shorter length of stay—outcomes that were shown to differ between the cohorts of our analysis. While Sweet et al [32] used matching, there were statistically significant differences in race and education between the 2 cohorts, which may have resulted from only using a 1:1 ratio, as opposed to the 1:3 ratio in this analysis. In contrast to our analysis, both Whaley et al [29] and Sweet et al [32] considered costs as the primary comparison metric and included different methodologies to calculate these costs, making them less applicable for comparison. Using event rates as the primary end point for comparison between DDS users and nonusers, as was done in this analysis, is a more objective measure.

It is also important, in line with the triple aim of the Institute for Healthcare Improvement, to consider how an intervention can improve the experience of care, improve the health outcomes of populations, and reduce per capita costs of health care as well as consider how it may contribute to health care provider burden, as introduced in the proposed quadruple aim [33,34]. A fifth point, advancing health equity, has been suggested to create a quintuple aim [35]. Given the evidence of reduced HCRU associated with DDS use provided by this study, the evidence of its clinical effectiveness, and the size of the population with T2DM in the United States, the DDS could make a substantial impact in helping to achieve the aims of the Institute for Healthcare Improvement [20,36].

This study was designed with the aim of combining the elements of observational studies (ie, real-world data and high generalizability) with a balance between cohorts typically seen in randomized controlled trials. This was achieved by using deidentified DDS data linked to extensive external real-world data sources through Health Insurance Portability and Accountability Act (HIPAA)–compliant tokenization and a meticulous matching methodology that resulted in well-matched cohorts as well as providing a more complete digital picture of data captured at the point of care and between points of care. Potential bias related to user motivation was mitigated by using blood glucose test strips as a proxy. DDS users were less likely to use blood glucose test strips than nonusers, although in this subgroup analysis, we were unable to account for the use of test strips that were obtained through self-payment or sent directly to the user.

While efforts were made to minimize limitations, they are inherent to any retrospective study. One of the limitations was that medical claims data were administrative records of care and were subject to the coding behaviors of health care providers; therefore, they were subject to potential miscoding. Within this study, there was potential for gaps in data coverage; however, this was mitigated by requiring continuous representation of data in the database during the baseline period. In addition, the potential for residual confounding remained, as we only adjusted confounders based on the available data.

The results of this study show a positive association between use of the DDS and lower HCRU and charges, but further investigation is warranted to explore the cause of some of the observed outcomes, such as the factors underlying the difference in prescription costs between DDS users and nonusers. In addition, further research into DDS users could provide evidence of their care-seeking patterns and assess whether and how this differs from nonusers. Longer-term research into the impact of DDS on HCRU is important as T2DM is a chronic condition, and quantifying the lifetime impact can provide a more comprehensive picture of value. Any such long-term research should take into account the evolution of digital technologies [9].

### Conclusions

This retrospective cohort study of adults in the United States with a diagnosis of T2DM found that DDS users had lower all-cause HCRU rates (hospitalizations and ER visits) than nonusers receiving usual care, largely driven by significantly lower inpatient hospitalization. In addition, HCRU charges and estimated costs were shown to be lower for DDS users compared with nonusers. These study results show the potential benefits of a DDS on HCRU outcomes that may be generalizable to adults with T2DM in the United States who own a smartphone.

## Appendix

Multimedia Appendix 1 Cost adjustment to 2022 US dollars.

Multimedia Appendix 2 Cost-to-charge ratio.

Multimedia Appendix 3 Standardized mean difference.

## Abbreviations

CCR: cost-to-charge ratio
DDS: Dario Digital Diabetes Solution
DM: diabetes mellitus
ER: emergency room
GLM: generalized linear model
HbA_1c_: glycated hemoglobin
HCRU: health care resource use
HIPAA: Health Insurance Portability and Accountability Act
IDV: Integrated Dataverse
IRR: incidence rate ratio
OR: odds ratio
PPPY: per person per year
T2DM: type 2 diabetes mellitus

## Data Availability

The datasets generated or analyzed during this study are not publicly available due to restrictions related to patient privacy and institutional data use agreements but are available from the corresponding author on reasonable request.
