# Supplementary material for: Effects of a Digital Health Intervention for Adults With Type 2 Diabetes Mellitus on Health Care Resource Use and Health Care Charges in the United States: Retrospective Cohort Study
Source: J Med Internet Res. 2025 Nov 17;27:e67320. doi: 10.2196/67320 (PMC12670060; doi:10.2196/67320)
Supplement: Multimedia Appendix 1 [file jmir_v27i1e67320_app1.docx]

## Supplementary Materials

### Cost Adjustment to 2022 Dollars

| Year | | Change From Previous Year (%) | Formula for Yearly CPI Factor to Convert to 2022 Dollars | CPI Factor |
| --- | --- | --- | --- | --- |
| **Medical (outpatient and office visits)** | | | |  |
|  | 2016 |  | =1.018*1.02*1.046*1.018*1.022*1.04 | 1.175199634 |
|  | 2017 | 1.8 | =1.02*1.046*1.018*1.022*1.04 | 1.154420072 |
|  | 2018 | 2 | =1.046*1.018*1.022*1.04 | 1.131784385 |
|  | 2019 | 4.6 | =1.018*1.022*1.04 | 1.08201184 |
|  | 2020 | 1.8 | =1.022*1.04 | 1.06288 |
|  | 2021 | 2.2 | =1.04 | 1.04 |
|  | 2022 | 4 | =1 | 1 |
| **Hospital services (inpatient and ER)** | | | |  |
|  | 2016 |  | =1.051*1.037*1.029*1.031*1.033*1.046 | 1.249359773 |
|  | 2017 | 5.1 | =1.037*1.029*1.031*1.033*1.046 | 1.188734323 |
|  | 2018 | 3.7 | =1.029*1.031*1.033*1.046 | 1.146320466 |
|  | 2019 | 2.9 | =1.031*1.033*1.046 | 1.114014058 |
|  | 2020 | 3.1 | =1.033*1.046 | 1.080518 |
|  | 2021 | 3.3 | =1.046 | 1.046 |
|  | 2022 | 4.6 | =1 | 1 |

CPI, Consumer Price Index; ER, emergency room.
