# Supplementary material for: Effects of a Digital Health Intervention for Adults With Type 2 Diabetes Mellitus on Health Care Resource Use and Health Care Charges in the United States: Retrospective Cohort Study
Source: J Med Internet Res. 2025 Nov 17;27:e67320. doi: 10.2196/67320 (PMC12670060; doi:10.2196/67320)
Supplement: Multimedia Appendix 2 [file jmir_v27i1e67320_app2.docx]

## Supplementary Materials

### Cost-to-Charge Ratio (CCR)

A CCR was used to convert charged amounts to costs. Total medical cost is defined from the payer’s perspective (total reimbursed amount) not from a provider’s perspective (total charged amount). Integrated Databerse (IDV) medical claim provides total billed amounts only. Remittance data can provide physician reimbursement by the plan and patient out of pocket costs. We linked the remittance date with IDV medical claim data to find or estimate the cost. Because only some of IDV medical claims could be matched to remittance data, we used the CCR calculated from remittance data and applied the CCR to the IDV medical claims that lack data on actual amount paid. This methodology was applicable to medical claims only. Paid pharmacy costs were directly obtained from the pharmacy claims information.

To estimate the paid amount for all medical claims available in IDV, we developed an average CCR for each billed service among a sample of patients for whom paid costs are available through remittance data.

***Methodology***

Step 1. Identify patients with type 2 diabetes mellitus (T2DM) who satisfy the inclusion and exclusion criteria defined in section 6.3 (DDS users and DDS non-users) and who have medical claims in the remittance data between January 1, 2016 and June 30, 2022.

Step 2. For each procedure code, by year, payer, payer type (primary, secondary, tertiary), and place of service, calculate the CCR using claim line paid amount (paid by payer) divided by claim line charged amount (billed).

Charged ≥ Allowed = Paid by Payer + Paid by Patient (co-pay/coinsurance)

Step 3. Produce a table of statistics by year, payer, payer type, and place of service, averaging across procedure category (Anesthesia, Surgery, Radiology Procedures, Pathology and Laboratory Procedures, Medicine Services and Procedures, Evaluation and Management Services, Category II Codes, Multianalyte Assay, Category III Codes) for each procedure code found in the data:

***Verify the Cost-to-Charge Ratio***

Step 4. Calculate total medical costs that were paid by insurance at the patient-level by summing paid amounts (claim line paid amount: amount the Payer reimbursed the provider after all adjustments) across all patient’s claims in the remittance data.

Step 5. Calculate estimated total paid costs by applying the CCR to remittance claims by multiplying the charged amount on the medical claim by the average CCR in the table above that corresponds to the procedure category, year, and payer on the claim to yield estimated insurance-paid amount for the service.

Step 6. Compare total paid costs in Step 4 to the calculated paid cost in Step 5 through descriptive statistics (mean, SD, median, first quartile (Q1) to third quartile(Q3)). Describe sources separately and calculate each patient’s difference in cost between the two methodologies.

***Apply the Cost-to-Charge Ratio***

Step 7. Once the CCR has been validated within patients with T2DM who have remittance data, the CCR can be applied to the larger patient population to obtain estimated paid medical costs for all services incurred in a 12-month period.

Step 8. Adjust costs for inflation using the consumer price index and convert all costs to 2022 US dollars.
