# Supplementary material for: Effects of a Digital Health Intervention for Adults With Type 2 Diabetes Mellitus on Health Care Resource Use and Health Care Charges in the United States: Retrospective Cohort Study
Source: J Med Internet Res. 2025 Nov 17;27:e67320. doi: 10.2196/67320 (PMC12670060; doi:10.2196/67320)
Supplement: Multimedia Appendix 3 [file jmir_v27i1e67320_app3.docx]

## Supplementary Materials

### Standardized Mean Difference

|  | | Unmatched | | | Matched | |
| --- | --- | --- | --- | --- | --- | --- |
| Variable | | Mean Difference | SD | Standardized Difference | Mean Difference | Standardized Difference |
| Logit propensity score | | 0.72886 | 0.83039 | 0.8772 | 0.00034 | 0.0004 |
| Age | | –6.08103 | 11.79683 | –0.51548 | –0.10093 | –0.00856 |
| Gender | | 0.02376 | 0.49941 | 0.04757 | 0.00006 | 0.00013 |
| **HCRU** | | | | | | |
|  | Inpatient | –0.0892 | 0.80433 | –0.1109 | 0.0241 | 0.02997 |
|  | ER | –0.11305 | 1.22828 | –0.09204 | 0.01986 | 0.01617 |
|  | Office visits | 0.24143 | 13.83367 | 0.01745 | 0.14504 | 0.01048 |
|  | Outpatient | –1.51796 | 12.30363 | –0.12337 | 0.0955 | 0.00776 |
| **Comorbidities** | | | | | | |
|  | Anemia | 0.03047 | 0.23738 | 0.12837 | –0.00286 | –0.01204 |
|  | Hypertension | 0.05017 | 0.48362 | 0.10374 | 0.00731 | 0.01511 |
|  | Hyperlipidemia | 0.02101 | 0.4988 | 0.04211 | 0.01016 | 0.02037 |
|  | Depression | –0.02177 | 0.3169 | –0.06869 | –0.00312 | –0.00984 |
| **Comedications** | | | | | | |
|  | Anxiolytics | –0.09919 | 0.47804 | –0.20749 | –0.00472 | –0.00987 |
|  | Anemia | 0.0086 | 0.13506 | 0.06364 | 0.00123 | 0.0091 |
|  | Hypertension | 0.04039 | 0.3943 | 0.10243 | 0.0102 | 0.02586 |
|  | Steroids | –0.015 | 0.45376 | –0.03306 | –0.00364 | –0.00802 |
|  | Dyslipidemia | 0.03632 | 0.46269 | 0.0785 | 0.00623 | 0.01346 |
|  | PPI | 0.0093 | 0.45066 | 0.02065 | –0.00201 | –0.00445 |

ER, emergency room; HCRU, health care resource utilization; PPI, proton pump inhibitor; SD, standard deviation.
